# Expression of JAK3, STAT2, STAT4, and STAT6 in pemphigus vulgaris

**DOI:** 10.1007/s12026-020-09122-y

**Published:** 2020-03-13

**Authors:** K. Juczynska, A. Wozniacka, E. Waszczykowska, M. Danilewicz, M. Wągrowska-Danilewicz, Agnieszka Zebrowska

**Affiliations:** 1 Department of Dermatology and Venereology, Medical University of Lodz, 1 Hallera Sq. Build. 6, 90-647 Lodz, Poland; 2 Department of Pathomorphology, Medical University of Lodz, Lodz, Poland; 3 Department of Nephropathology, Medical University of Lodz, Lodz, Poland

## Introduction

Pemphigus vulgaris (PV) is an autoimmune intraepidermal bullous disease with a prevalence of 0.1–0.7 per 100,000 individuals [1]. Clinical demonstration of the disease includes flaccid blisters and erosions present within both skin and mucous membranes [2]. Characteristics of the PV disease are IgG deposits localized within lesional epidermis revealed in direct immunofluorescence examination (DIF) and circulating autoantibodies present in indirect immunofluorescence (IIF). The target antigens are cadherin molecules: desmoglein (DSG) 3 and DSG 1 [3]. After attachment of antibodies to antigens, several synergistic processes take place leading to loss of cell adhesion [4]. It is suggested that both signaling-dependent and signaling-independent pathways contribute to acantholysis development [5]. All those processes occur with participation of various cytokines and chemokines, wherein elevated levels were detected in serum, blister fluid, and perilesional skin of patients with PV. Moreover, levels of some of them were found correlating with activity of PV [6].

The Janus kinases (JAKs) and signal transducers and activators of transcription (STATs) are a family of proteins constituting signaling pathway. In mammals, the STAT family includes seven members (STAT1, STAT2, STAT3, STAT4, STAT5a, STAT5b, STAT6) and four tyrosine kinases (JAK1, JAK2, JAK3, and TYK2) [7, 8]. The cascade might be activated by numerous signaling molecules; it enables intercellular communication and plays a significant role in proliferation, growth, differentiation, migration, and apoptosis of cells [9]. There have been inflammatory and autoimmune diseases identified where the JAK/STAT signaling is disrupted [10, 11]. However, there are no reports concerning the JAK/STAT pathway and its contribution to pathogenesis of pemphigus vulgaris yet to be published.

Thus far, literature data seem to indicate an influential role of JAK/STAT in the pathogenesis of autoimmune skin disorders, with IL-4, INF, TNF-α, IL-6, IFNs, and IL-17 being key mediators [10, 11]. Specifically, the JAK/STAT pathway is instrumental for the Th2 cell differentiation [9]. That is why the aim of this study was to evaluate the expression of proteins JAK3, STAT2, STAT4, and STAT6 in skin lesions and perilesional area in patients with pemphigus vulgaris as well as in the control group.

## Materials and methods

### Patients

The study included 15 persons with PV (11 women and 4 men; range 59 to 89 years, average 72.51 years). All patients were at an active stage of the disease, before administration of any (systemic or topical) treatment. The control group comprised 10 healthy, unrelated volunteers, matched for sex and age. Skin samples of healthy volunteers have been taken from similar areas of those of disease’s groups. Diagnosis of PV was established based on medical history, clinical picture, and immunofluorescence findings.

Before entering the study, all the patients gave their informed written consent. The study protocol RNN/132/07/KB was approved by the Local Ethical Committee of the Medical University of Lodz.

### Methods

Immunohistochemical methods were used to evaluate expression of JAK3, STAT2, STAT4, and STAT6 in both lesional and perilesional skin, and compared with healthy control skin. Paraffin-embedded tissue sections were mounted onto SuperFrost slides, deparaffinized, then treated in a solution of TRS and transferred to distilled water. Endogenous peroxidase activity was blocked by 0.3% hydrogen peroxide in distilled water and then, sections were rinsed with Tris-buffered saline (TBS, Dako, Denmark), and incubated with primary rabbit polyclonal antibody against STAT2 (Santa Cruz Biotechnology Inc.), mouse monoclonal antibody against STAT4 (Santa Cruz Biotechnology, Inc.), and primary rabbit polyclonal antibody against STAT6 (Santa Cruz biotechnology Inc.) and incubated overnight with mouse monoclonal antibody against JAK3. Immunoreactive proteins were visualized using EnVision-horseradish peroxidase kit (Dako, Carpinteria, CA, USA) according to the instructions of the manufacturer. Visualization was performed by incubation of the sections in a solution of 3,3′-diaminobenzidine (DakoCytomation, Denmark). After washing, the sections were counter-stained with hematoxylin and coverslipped. For each antibody and for each sample, a negative control was processed.

### Semiquantitative analysis

Expression was evaluated according to methodology derived from research by Tam et al. [12]. In each specimen, staining intensity of JAK3, STAT2, STAT4, and STAT6 was recorded semiquantitatively by two independent observers in 7–9 high-power fields using in each field a weighted histoscore method according to Kirkegaard et al. (2006), also known as the H score system [13]. The immunoexpression score was calculated as follows (1 × % cells staining weakly positive) + (2 × % cells staining moderately positive) + (3 × % cells staining strongly positive). The mean score for each specimen was calculated by averaging grades assigned by the two authors and approximating the arithmetical mean to the nearest unity. All values were expressed as the mean ± SD (standard deviation).

## Results

**Fig. 1:**
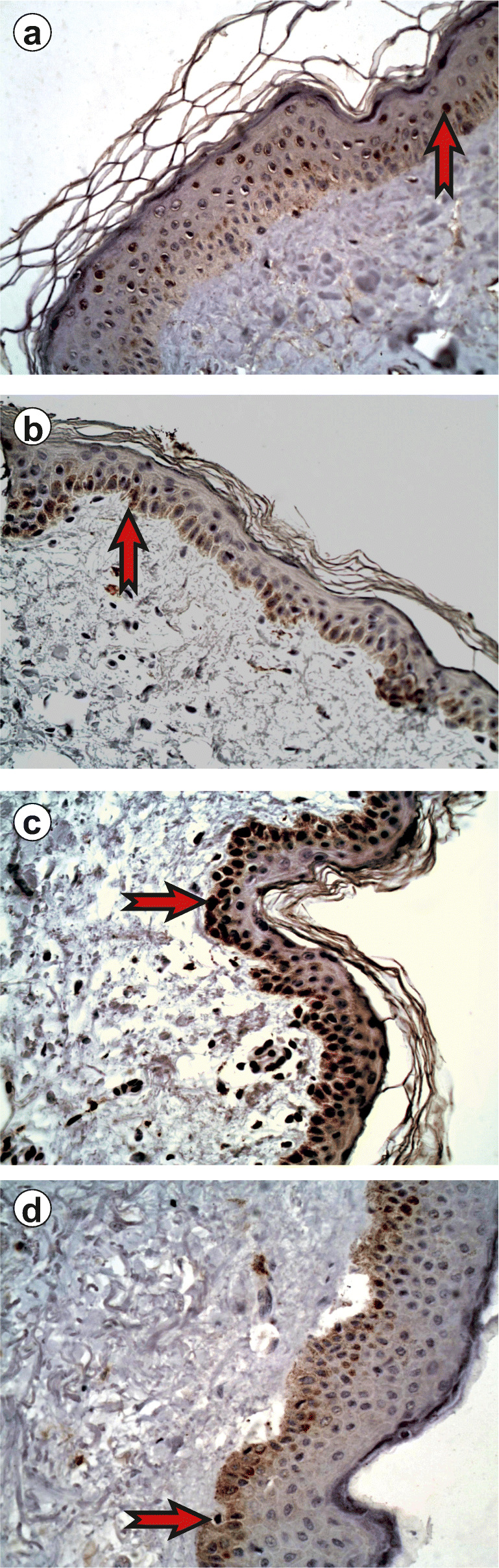
Immunoexpression of JAK/STAT proteins in the epidermis, normal skin, × 400. **a** Immunoexpression of JAK3 in the epidermis, normal skin, 10.73 ± 3.36. **b** Immunoexpression of STAT2 in the epidermis, normal skin, 11.06 ± 5.34. **c** Immunoexpression of STAT4 in the epidermis, normal skin, 18.59 ± 3.01. **d** Immunoexpression of STAT6 in the epidermis, normal skin, 11.56 ± 2.84

**Fig. 2:**
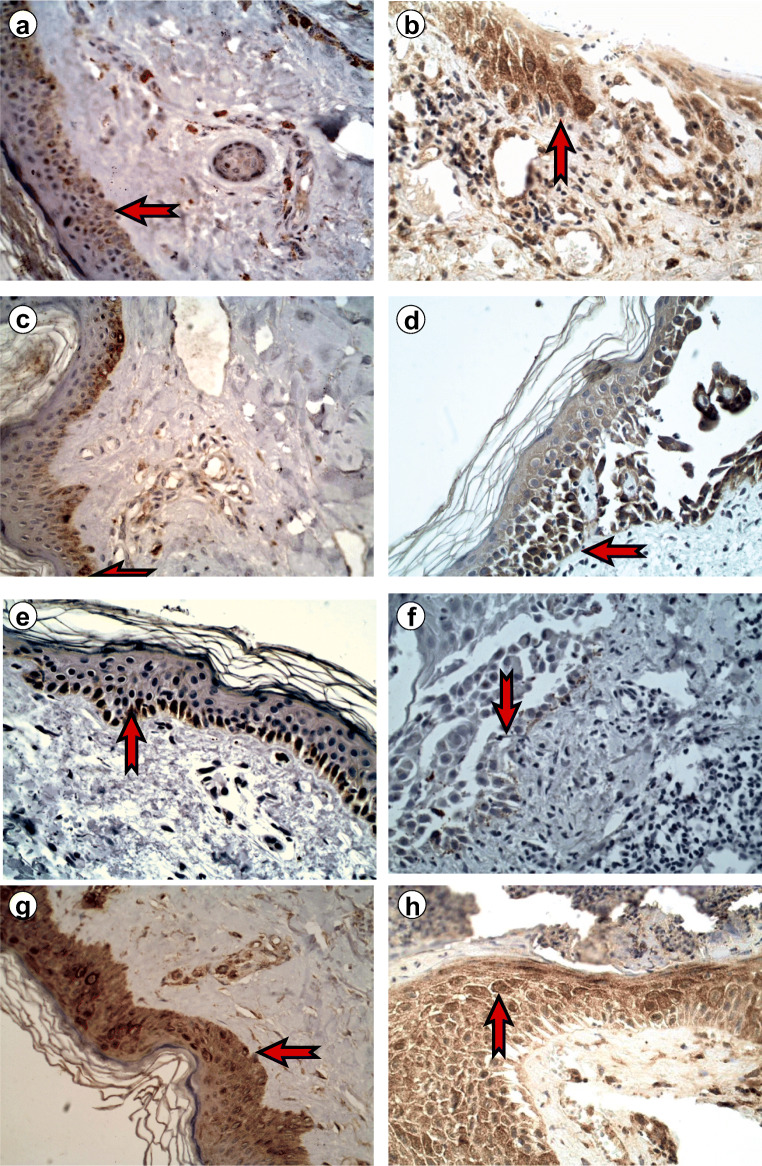
Immunoexpression of JAK/STAT proteins in the epidermis, pemphigus vulgaris, × 400. Immunoexpression of JAK3 in the epidermis: **a** perilesional skin 14.38 ± 3.61; **b** skin lesions 18.89 ± 4.67, *p* > 0.05. Immunoexpression of STAT2 in the epidermis: **c** perilesional skin 15.79 ± 2.06; **d** skin lesions 17.15 ± 2.81, non-significant. Immunoexpression of STAT4 in the epidermis: **e** perilesional skin 24.10 ± 3.40; **f** skin lesions 29.08 ± 4.38, *p* < 0.05. Immunoexpression of STAT6 in the epidermis: **g** perilesional skin 18.21 ± 3.49; **h** skin lesions 27.85 ± 4.68, *p* < 0.05

In healthy skin samples, expression of JAK3 was found throughout the epidermis with the horny layer being strongly stained by the antibody against JAK3. Immunoreactivity of STAT2, STAT4, and STAT6 antibodies was more strongly detected in the granular layer than in lower layers of epidermis. The horny cell layer was not stained with the antibodies against STAT2, STAT4, and STAT6 (Figs. 1 and 2).

Expression of JAK3 was significantly higher in PV skin lesions (17.04 ± 4.16) in comparison with the control group (10.73 ± 3.36; *p* < 0.05) and perilesional skin (13.18 ± 3.81; *p* > 0.05). There was no significant difference (*p* > 0.05) between JAK3 expression in PV perilesional skin and the control group.

Expression of STAT2 was higher in PV patient skin lesions (19.15 ± 1.70) than in the control group (11.06 ± 5.34; *p* < 0.05). There was also statistical difference evaluated between PV skin lesions and perilesional area (13.91 ± 2.61; *p* < 0.05), but there was no statistical significance between expression of STAT2 in PV perilesional area and the control group (*p* > 0.05).

There was no significant difference between expression of STAT4 in PV skin lesion (19.37 ± 1.69) and the control group (18.59 ± 3.01; *p* > 0.05), as well as between PV skin lesions and PV perilesional skin (19.69 ± 3.23; *p* > 0.05). There was also no statistical difference between expression of STAT4 in skin lesions and perilesional area in patients with PV (*p* > 0.05).

**Fig. 3:**
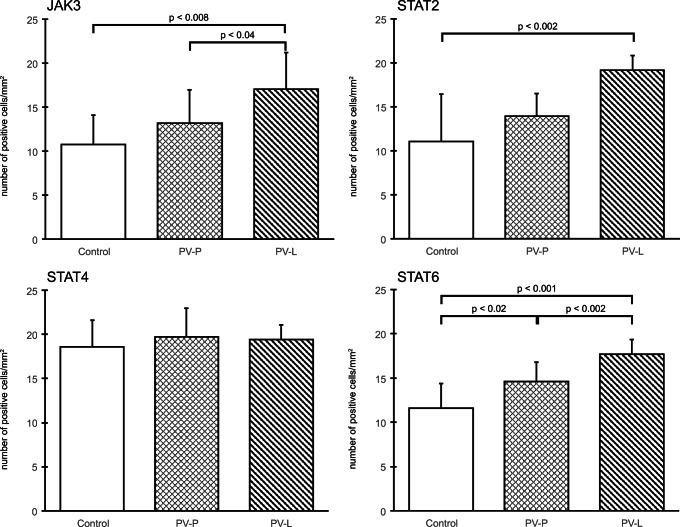
Morphometric analysis of JAK3 (upper left), STAT2 (upper right), STAT4 (lower left), and STAT6 (lower right) immunoexpression in keratinocytes. The results of semiquantitative analysis are expressed as the mean ± standard deviation. Control – normal skin. PV-P – pemphigus vulgaris perilesional skin, PV-L – pemphigus vulgaris skin lesions. The level of significance is defined where *p* < 0.05. JAK3: C vs. PV-L (*p* < 0.05); PV-P vs. PV-L (*p* < 0.05); STAT2: C vs. PV-L (*p* < 0.05); STAT4: C vs. PV-P (NS), C vs. PV-L (NS); STAT6: C vs. PV-P (*p* < 0.05), C vs. PV-L (*p* < 0.05), PV-P vs. PV-L (*p* < 0.05)

The medium intensity of STAT6 expression was higher in PV skin lesions (17.65 ± 1.64) as compared with that in the control group (11.56 ± 2.84; *p* < 0.05). There was also significant difference between STAT6 expression in PV skin lesions and perilesional PV skin (14.48 ± 2.17; *p* < 0.05), as well as between healthy skin and PV perilesional skin (*p* < 0.05) (Fig. 3).

## Discussion

There are many researches investigating the activation of JAKs and STATs by particular signaling molecules. TNF-α, IL-6, IFNs, IL-17, IL-21, and IL-23 among others are known for their contribution to inflammatory process also in the course of pemphigus [8]. As explicit above, they are able to use JAK/STAT pathway to transmit their signal, especially JAK3 and STAT4, STAT6, and STAT1. That is the reason of choosing these proteins to examine in PV.

Immunohistochemical research by Nishio et al. [14] revealed higher expression of JAK2, JAK3, STAT1, and STAT5 in the horny cell layer, and abundant expression of JAK3, TYK2, STAT2, STAT3, STAT4, and STAT6 in the granular layer of the epidermis in healthy volunteers. Our findings in the control group reveal differential expression and immunolocalization of JAK3, STAT2, STAT4, and STAT6 in healthy human epidermis, and are consistent with the results by Nishio et al. It is suggested, that JAK/STAT pathway may play an important role in keratinocyte differentiation [15].

The JAK/STAT signaling pathway might be activated by numerous signaling cytokines, growth factors, and hormones, such as interferon-α/β/γ (IFN-α/β/γ), IL-2, IL-4, IL-6, IL-7, IL-9, IL-10, IL-12, IL-15, IL-19, IL-20, IL-21, IL-22, and IL-23, erythropoietin (Epo), granulocyte colony-stimulating factor (G-CSF), epidermal growth factor (EGF), and platelet-derived growth factor (PDGF) [16, 17]. Attachment of the signaling molecule to its transmembrane receptor is the first step of signaling cascade; then, activation of the associated with the receptor JAK takes place. After activation, JAKs phosphorylate cytokine receptors, what enables STAT monomers to bind to the complex and form homo- and heterodimers due to tyrosine phosphorylation. Activated STATs translocate to the cell nucleus and bind to DNA, what enables transcription of target genes [18].

In the pathomechanism of PV, both humoral and cell-mediated immune response are involved. It is reported that PV patients show an increased expression of numerous cytokines and chemokines. Literature data indicates that elevated levels of IL-1β, IL-4, IL-6, IL-8, IL-10, IL-12, IL-15, and tumor necrosis factor α (TNF-α) were found in the serum and/or blister fluid of patients with PV [18, 19]. Data concerning Th1 and Th17 pathway are inconsistent, showing contrary levels of IL-2, IFNγ, and IL-17 present in PV patients, what makes Th1 and Th17 pathways’ role in PV pathogenesis still an open issue [19].

It is crucial to underline that obtained results concerning particular cytokine levels are equivocal in majority of analyzed studies. Up to date studies examining cytokine levels in PV patients have several limitations. First of all, the number of patients included in researches is relatively small. Moreover, serum and tissue samples were collected at various points of the disease course [20]. Although widely examined, this issue requires further studies.

STAT4 and STAT6 are both strongly involved in inflammatory processes, as they are activated by proinflammatory cytokines, such as IL-12, IFNγ (both activate STAT4) and IL-4, IL-13 (activating STAT6) [21]. It is reported that JAK2 and JAK1, JAK3 and STAT6, as a IL-4 signaling components, are critical for Th1 cell differentiation, and JAK1 and 3/STAT6, as a IL-4 signaling pathway, are essential for Th2 differentiation [22]. Our results suggest that STAT6 may contribute to PV pathogenesis, probably due to its contribution to Th2 immune responses. This results stay in accordance with the majority of published researches, suggesting Th2-pathway upregulation and elevated serum levels of IL-4, IL-6, and IL-10 in PV patients [23]. Moreover, IL-6 is one of the cytokines of which level is shown to be correlating with PV severity, what underlines meaning of STAT6 upregulation in PV [24].

Balance disturbance in Th1/Th2 response in PV pathomechanism has been widely reported [25]. Lee at al. [26] reported decreased level of IFNγ and supported suppressed Th1 response in PV active stage. However, in literature, there are also contradictory results concerning Th1 pathway and its role in PV pathogenesis present. Results by Timoteo et al. [27] showing increased IFNγ serum level suggest that PV induces Th1 immune response. Our results follow studies showing no relevant differences in Th1 cytokine serum levels as compared with control group [6]. In our research, estimated expression of STAT4, high but similar in every examined sample, indicates that this protein does not participate in pathogenesis of PV.

As reported, STAT2 is involved in transmitting signal initiated mainly by interferons [28]. The expression of STAT2, significantly higher in PV skin lesions than in healthy skin, suggests participation of STAT2 and cytokines activating it (interferons α and β) in pathogenesis of the disease. Interferons α and β activate JAK1 and TYK2 kinases, and subsequently STAT1 and STAT2 proteins, while IFNγ requires JAK1 and JAK2 kinases and STAT1 homodimers in signal transmitting. As mentioned, interferons use JAK/STAT pathway to transmit their signals as well. Interferons α and β activate JAK1 and TYK2 kinases, and subsequently STAT1 and STAT2 proteins, while IFNγ uses JAK1 and JAK2 kinases and STAT1 homodimers in transmitting signal [28].

The results of our research suggest that STAT2 may be involved in proinflammatory reactions taking place in both PV lesional and perilesional areas. The contribution of STAT2 to PV pathogenesis needs to be further examined.

Reports concerning transmitting IL-17 signal by STAT proteins are equivocal [29]. However, results of numerous researches indicate that JAK3/STAT3 pathway mediates IL-23 and IL-6 dependent signals and implicates the Th17 cell differentiation, and hence indirectly promotes IL-17A transcription [30]. IL-17A is able to induce Th2 cell activation, directly recruit neutrophils and eosinophils, and produce cytokines and metalloproteinases [31]. JAK3 also acts on receptors with common γ chain (IL-2, IL-4, IL-7, IL-9, IL-15, and IL-21), crucial to lymphocyte maturation and function [32].

There are single reports concerning role of Th17 cells in PV pathogenesis. Arakawa et al. [33] reported presence of Th17 cells in PV skin lesions, but without correlation with disease activity or anti-DSG3 antibody level. On the other hand, Giordano et al. and Timoteo et al. [27] reported increased level of IL-17A in PV patients as compared with control. However, results by Lee et al. and Masjedi et al. indicated no significant difference in IL-17A level between PV and healthy control. [26].

Expression of JAK/STAT proteins in PV and their suggested role in pathogenesis of the disease create new potential therapeutic targets for the treatment of blistering diseases. JAK inhibitors have shown positive clinical results in skin diseases, like psoriasis, alopecia areata, and allergic dermatitis among others [29].

The presented study is the first one exploring the expression of JAK/STAT proteins in pemphigus. It would be of great importance to examine expression of all JAK/STAT proteins in PV patients and compare findings with results obtained in a comparable research.

Undoubtedly, the examined issue brings prospects for future studies. The confrontation of particular JAK/STAT expressions and cytokine levels in serum and blister fluid could give more detailed data on the role of examined proteins and cytokines in PV pathogenesis. However, this issue requires further studies, as a target for new therapeutic agents as well.
